# Supplementary material for: Assessment of transmitral and left atrial appendage flow rate from cardiac 4D-CT
Source: Commun Med (Lond). 2023 Feb 11;3:22. doi: 10.1038/s43856-023-00252-6 (PMC9922288; doi:10.1038/s43856-023-00252-6)
Supplement: Supplementary file 3 — Supplementary Information [file 43856_2023_252_MOESM3_ESM.pdf]

Assessment of transmitral and left atrial appendage flow rate from cardiac 4D-CT

Supplementary material

Sophia Bäck, Lilian Henrikson, Ann F Bolger, Carl-Johan Carlhäll, Anders Persson, Matts Karlsson, Tino Ebbers

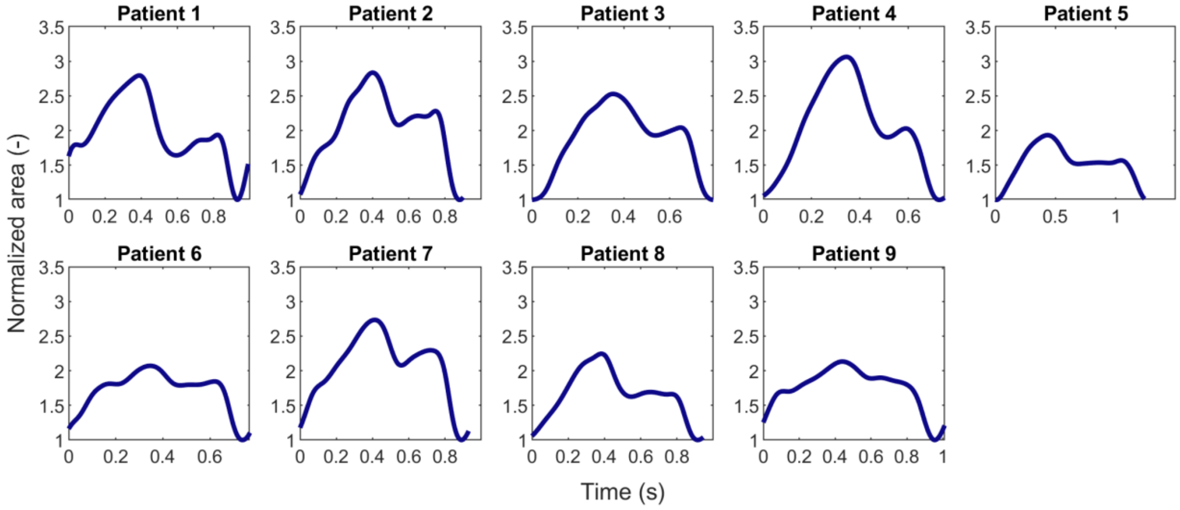

Supplementary Figure 1: Normalized surface area of the LAA over time

Supplementary Figure 1 shows the normalized surface area of the LAA over time, calculated according to equation 1:

$$A_{norm}(t) = \frac{A_{LAA}(t)}{A_{LAA\_min}} \quad (1)$$

Where A_{LAA} is the time dependent surface area of the LAA and A_{LAA_min} the subject specific minimal LAA surface area. The surface area was calculated based on a segmentation at the first time step, which was then deformed according to the motion tracking results. The maximum increase of surface area differs between individuals. For patient 5, the area doubles during the cardiac cycle, while it triples for patient 4.

Al-Issa et al[1]. described the change in LAA surface area using the averaged area change ratio ($\overline{\%AC}$). To be able to compare this to our results, we derived the normalized area from the averaged area change ratio according to equation 2:

$$A_{norm} = 1 + \frac{\overline{\%AC}}{100} \quad (2)$$

Both Otani et al. [2] and Al-Issa et al. used the LAA surface area at 0%RR as the reference surface. In their data, this corresponded to the minimal LAA surface area. In our data, the minimal LAA surface area was often at the end of the cardiac cycle, around 95%RR.

Supplementary References

1. Al-Issa, A. *et al.* Regional function analysis of left atrial appendage using motion estimation CT and risk of stroke in patients with atrial fibrillation. *Eur. Heart J. Cardiovasc. Imaging* **17**, 788–796 (2016).
2. Otani, T., Shiga, M., Endo, S. & Wada, S. Performance assessment of displacement-field estimation of the human left atrium from 4D-CT images using the coherent point drift algorithm. *Comput. Biol. Med.* **114**, 103454 (2019).
