# Supplementary figures and images for: Assessment of transmitral and left atrial appendage flow rate from cardiac 4D-CT
Source: Commun Med (Lond). 2023 Feb 11;3:22. doi: 10.1038/s43856-023-00252-6 (PMC9922288; doi:10.1038/s43856-023-00252-6)

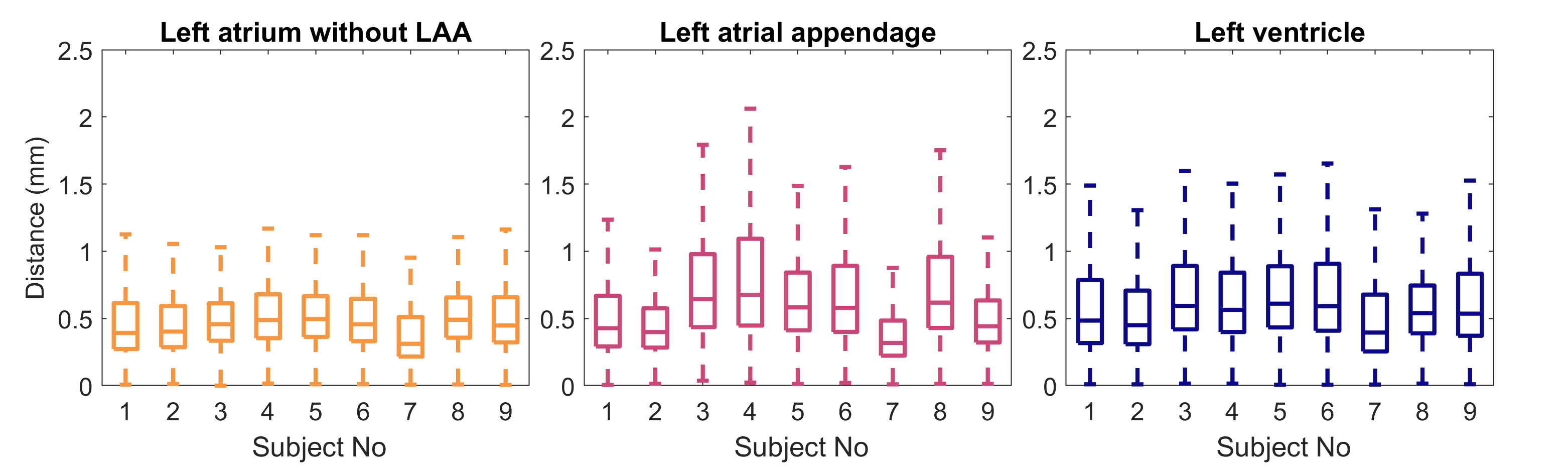

Supplement: Supplementary file 4 — Supplementary Data 1 [file 43856_2023_252_MOESM4_ESM.zip › SupplementaryData1/Figure2_DistanceToTarget/Registration_distance_Boxplot.png]

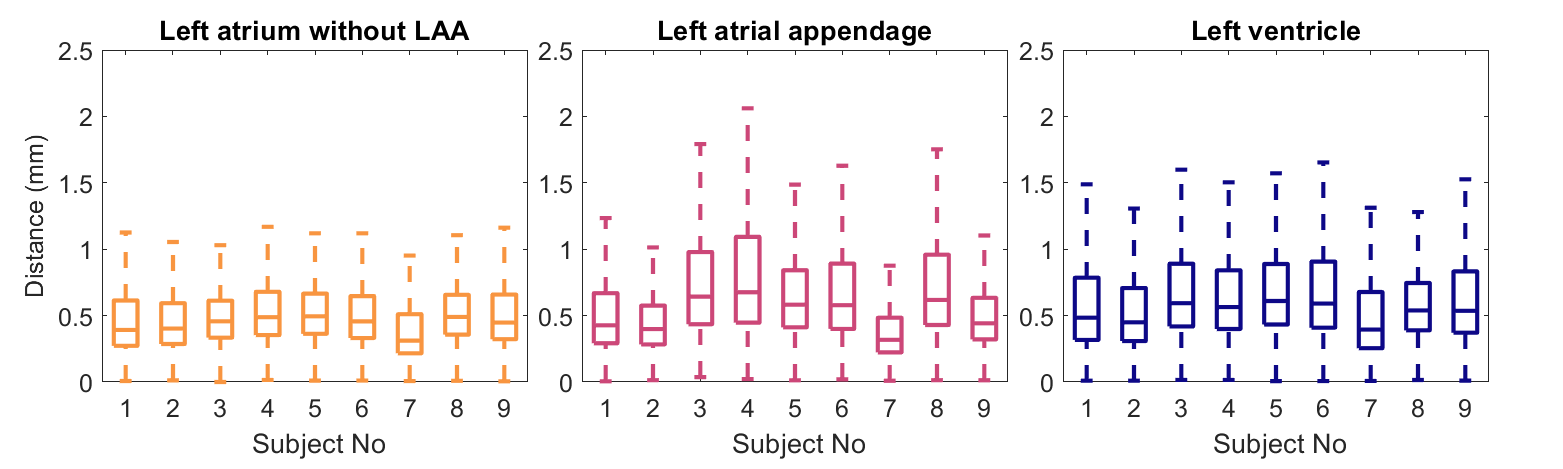

Supplement: Supplementary file 4 — Supplementary Data 1 [file 43856_2023_252_MOESM4_ESM.zip › SupplementaryData1/Figure2_DistanceToTarget/Registration_distance_Boxplot.tif]
