# Supplementary material for: Assessment of transmitral and left atrial appendage flow rate from cardiac 4D-CT
Source: Commun Med (Lond). 2023 Feb 11;3:22. doi: 10.1038/s43856-023-00252-6 (PMC9922288; doi:10.1038/s43856-023-00252-6)
Supplement: Supplementary file 5 — Description of Additional Supplementary Files [file 43856_2023_252_MOESM5_ESM.pdf]

Description of Additional Supplementary Files

File name: Supplementary Data 1

Description: All source data underlaying the graphs and charts presented in the main and supplementary figures

File name: Supplementary Movie 1

Description: Animation of distance between registration and target surface
